# Trails of ants converge or diverge through lens-shaped impediments, resembling principles of optics

**DOI:** 10.1038/s41598-020-65245-0

**Published:** 2020-05-21

**Authors:** Jibeom Choi, Hangah Lim, Woncheol Song, Han Cho, Ho-Young Kim, Sang-im Lee, Piotr G. Jablonski

**Affiliations:** 1 Laboratory of Behavioral Ecology and Evolution, School of Biological Sciences, Seoul National University, Seoul, 08826 South Korea; 2 College of Medicine, Seoul National University, Seoul, 03080 South Korea; 3 Department of Mechanical and Aerospace Engineering, Seoul National University, Seoul, 08826 South Korea; 4 Laboratory of Integrative Animal Ecology, Department of New Biology, DGIST, Daegu, 42988 South Korea; 5 Museum and Institute of Zoology, Polish Academy of Sciences, Warsaw, 00-679 Poland

**Keywords:** Behavioural ecology, Animal behaviour

## Abstract

Analogies across disciplines often indicate the existence of universal principles such as optimization, while the underlying proximate mechanisms may differ. It was reported recently that trails of ants refract at the border of substrates, on which walking speeds differ. This phenomenon is analogous to the travel-time-minimizing routes of light refracting at the borders between different media. Here, we further demonstrate that ant tracks converge or diverge across lens-shaped impediments similar to light rays through concave or convex optical lenses. The results suggest that the optical principle of travel time reduction may apply to ants. We propose a simple mathematical model that assumes nonlinear positive feedback in pheromone accumulation. It provides a possible explanation of the observed similarity between ant behavior and optics, and it is the first quantitative theoretical demonstration that pheromone-based proximate mechanisms of trail formation may produce this similarity. However, the future detailed empirical observations of ant behavior on impediment edges during the process of pheromone trail formation are needed in order to evaluate alternative explanations for this similarity.

## Introduction

Optimization is a central principle underlying a variety of physical, chemical, and biological phenomena^1^ including ecology and evolution of biological organisms^2,3^. It is possible that similar underlying principles of optimization may lead to similarities between biological and physical phenomena.

Optimization of foraging efficiency by individuals or groups (*e*.*g*. colonies of social insects) is a product of natural selection for optimal foraging^2^. One of the mechanisms involved in foraging optimization is the ability of animals to minimize their travel time during foraging trips^4–7^. For example, colonies of ants are able to establish foraging traffic in a time-minimizing manner: army ants *Eciton burchelli* are able to form the trails along which flow of the traffic is maximized^8^, and Argentine ants *Iridomyrmex humilis* can collectively form pheromone-marked foraging trails that reduce the travel time on bifurcated bridges^9^. Ants may reach such a colony-level optimization through interactions among individuals without central control over their behavior. One of the crucial processes of the trail formation includes individuals marking their travel path from the food source to the nest with a pheromone. This pheromone-marked path is later used by other foragers who travel between the nest and the food source adding their own pheromone to the trail to reinforce the path. Additional processes involved in the formation of foraging trails include path integration, spatial memory, or edge-following^10–15^. This ability of ants to optimize the collective performance through the decentralized processes at the level of an individual has inspired other fields of science such as robotics and computer science^16,17^.

The principle of reduced travel time also governs the behavior of light rays. According to Fermat’s least-time principle, which is an axiomatic principle in optics, a ray of light travels from a certain point to another through a time-minimizing trail. In optics, the time-minimizing path does not need to be distance-minimizing. When a light ray traverses across a border between two media of different light propagation speeds (different refractive indices), it bends (refracts) in such a manner that minimizes the light’s travel time through the two media. Snell’s law, or the law of refraction, was derived from Fermat’s principle in order to compute the angles of incidence and refraction that determine how a light ray bends at the edge between the two media. As both ants and light rays are governed by a similar principle of travel time minimization, it is expected that the pheromone-based travel-time-reducing trails laid by ants also bend at the edge between two substrates of different ant walking speeds. This prediction was recently tested in laboratory conditions in one species of ants^18^. It was shown that the little fire ants *Wasmannia auropunctata* entering a substrate that slows them down refract their trails at the border between substrates in a way similar to laws of optics^18^ resulting in time-reducing paths similar to the light rays. Experiments on *Lasius niger*^7^ suggested that preference for path of shorter travel time might be a consequence of behavioral and physical processes involved in the pheromone trail formation, and these processes were hypothesized as the proximate mechanisms responsible for the analogy between optics and ant behavior^7,18^.

**Figure 1:**
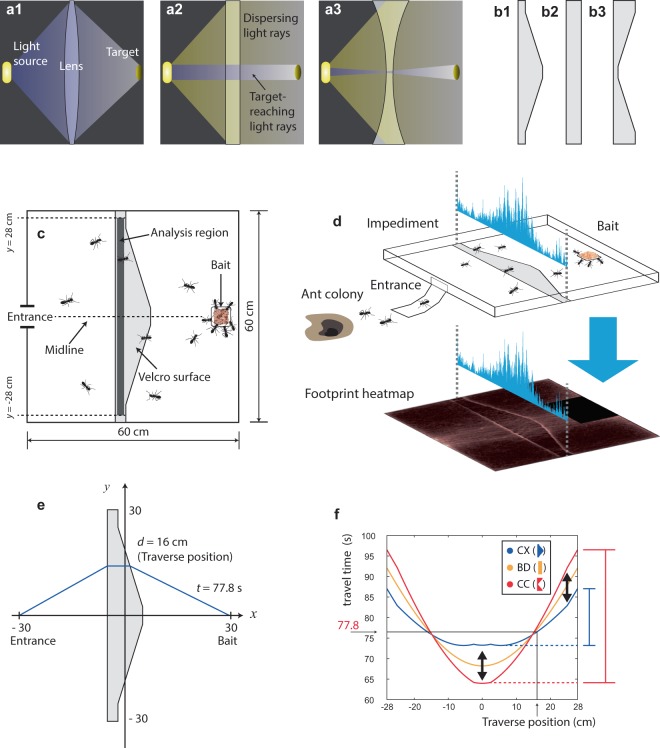
The schematics of light rays passing through optical lenses (**a1–a3**), the materials and methods used in the research (**b1–b3, c,d**), and the methods of mathematical analysis (**e,f**). (**a1–a3**)–Violet-shading represents light rays that reach the target through the lens, while yellow-shading represents those that cannot reach the target. For the convex lens (**a1**), if properly positioned, all target-reaching paths of light from the source are time-minimizing. For the flat plane (**a2**) and concave lens (**a3**), the target-reaching light rays form narrow beams. (**b1–b3**)–The shapes of convex-shaped (**b1**), band-shaped (**b2**), and concave-shaped (**b3**) impediments made of Velcro to reduce the walking speed of ants. (**c**)–Schematics of the experimental arena with a convex-shaped impediment. The impediment is marked with light gray, and the 2-cm-wide analysis region is marked with dark gray. The area within 2 cm from the walls of the experimental arena was exempted from the analysis. (**d**)–An example of the weighted relative frequency distribution of ant locations defined as the displacements from the midline (*y*-axis in (**e**); ranging from −28 to 28 cm). (**e**)–An example of a computed path linking the entrance and the bait through the impediment at a certain displacement (d) from the midline. Here, the path (blue line) crosses the impediment at d = 16 cm from the midline. Given that an ant’s average speed on the paper-lined surface is 1 cm/s and the average speed through the Velcro is 0.39 cm/s, it takes 77.8 s to travel the path whose traverse position is d = 16 cm. (**f**)–An example (Set 1, colony MS) of relationships between the displacement from the midline (traverse position) and the expected travel time calculated from the empirical values of ant walking speed. The travel time function for the convex-shaped impediment (blue line) is relatively flat compared to that of the concave-shaped impediments (red line) as shown by red and blue bars on the right side. The example path of (**e**) is marked with the traverse position. See Fig. S2 for other travel time functions.

Another optical rule, which can be derived from Fermat’s principle and Snell’s law, is the lensmaker’s equation. This equation predicts a focal distance of a thin lens from the refraction ratio and curvature of the lens. We focused on the fact that this equation also predicts whether the target-reaching light rays would diverge (wide dispersion along with the lens) or converge (narrow dispersion) across convex or concave lenses (violet-shaded light rays in Fig. 1a1–a3). If target-reaching light rays diverge or converge on lenses, we hypothesized (based on the general similarity between ant trails and laws of optics^18^) that bait-reaching trails of foraging ants would also follow patterns similar to the light rays across lenses, and that they would show higher dispersion across convex impediments that slow ants down (Fig. 1b1) and lower dispersion across concave impediments (Fig. 1b3) located between their nest and the food source (Fig. 1c). We attempted to investigate this general analogy between the effect of lens shape on the dispersion of target-reaching light rays (resulting from the physics of light; Fig. 1a1–a3) and the effect of impediment shape on the dispersion of ant foraging paths resulting from the collective behavior of individual ants. In order to do so, we observed trajectories of ants across passable lens-like impediments made of Velcro that slow the ants down (Fig. 1b1–b3). We used natural, rather than laboratory-based, ant colonies to minimize the possibility of behavioral artifacts. Additionally, we quantitatively explored the hypothesis that the processes involved in pheromone trail formation across the experimental impediments might have been responsible for the observed analogy between optics and ant behavior. We expressed this hypothesis quantitatively in a simple mathematical model that grasps the nonlinear positive feedback between the number of ants on a path and the pheromone accumulation.

## Results

### Experiments

We observed foraging tracks of the Japanese carpenter ant, *Camponotus japonicus*, across the passable impediments of three shapes: convex, band, and concave (Fig. 1b1–b3). The impediments were located between the nest and the feeder (‘Bait’ in Fig. 1c). We performed two sets of experiments, and each set comprised three treatments (impediment shapes) at three independent colonies, resulting in 18 recorded trials. From a frame-by-frame analysis of video of each trial, we calculated a relative frequency distribution (blue-colored distribution in Fig. 1d) of ants’ locations (defined by values of displacement from the midline, *y*_*i*_) weighted by each ant’s horizontal speed at each coordinate (*v*_*i,h*_*;* Fig. 1c,e) within the impediment’s analysis region (a dark band in Fig. 1c; see **Methods** for details). The weighted standard deviation ***S*** was used as an index of dispersion (See **Methods** for the detailed explanations) and it was compared to the expected dispersion. Additionally, in order to determine the expected dispersion, we used walking speeds of ants recorded in each trial to calculate the relationships between the displacement from the midline (*y*_*i*_) and the expected travel time (example in Fig. 1f; details and results for all tests are given in the Supplementary Materials).

**Figure 2:**
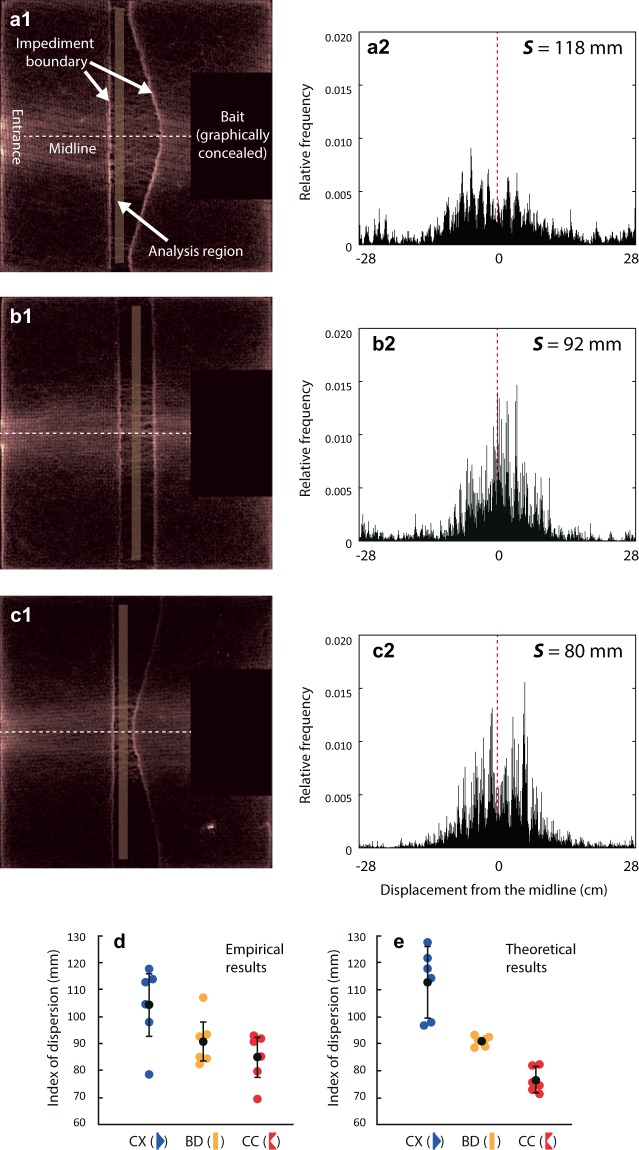
Examples of walking ants’ density heatmaps (**a1–c1**), the corresponding frequency distributions (**a2–c2**), and the index of dispersion, *S*, from empirical (**d**) and theoretical (**e**) results. (**a1–c1**)–All the positions of individual walking ants during the analysis period (60 min-90 min since the start of the experiment) are visualized in a heatmap, in which the brighter grids indicate the higher number of ants walking through the grid (1 mm × 1 mm; see Supplementary Methods for details). The bait and nearby regions (black-colored rectangles) are graphically concealed in order to exclude those ants that are stationary near the bait from the visualization. (**a2–c2**)–The corresponding relative frequency distributions based on the displacements from the midline of ants crossing the analysis region of the convex- (**a2**), band- (**b2**), and concave-shaped (**c2**) impediments, respectively. The data were obtained from tests of Set 1 at the colony MG. The frequency distributions across convex-shaped impediment exhibit the greatest divergence compared to those on band-shaped and concave-shaped impediments. (**d**)–The effect of impediment shape on the dispersion index, ***S***, in all 18 experiments. See Fig. S1 of the Supplementary Materials which is an alternative figure for the same data. (**e**)–The effect of impediment shape on the theoretical dispersion index (*Ŝ*) calculated for each experiment from the data of ants’ walking speeds on the paper-lined surface (*v*_*N*_) and on the Velcro surface (*v*_*V*_) shown in Table S1 in Supplementary Materials. In (**d**) and (**e**), the blue dots indicate the convex-shaped impediment treatment (CX), yellow dots, the band-shaped treatment (BD), and red dots, the concave-shaped (CC) impediment treatment. Black circles indicate the average value of ***S*** (or *Ŝ*) for each treatment, and error bars represent 95% confidence intervals. 95% CI for band-shaped impediment in (**e**) was too small to show (± 2 mm).

As predicted, the index of dispersion ***S*** varied by the shape of the impediments in our empirical data (Fig. 2a–d; mixed-model ANOVA, *F* = 6.661, *df* = 2, *p* = 0.011; nest ID and set ID were considered as random effects). ***S*** across the convex-shaped impediment (Fig. 2a1,d–blue dots) was significantly higher than ***S*** across the band-shaped (Fig. 2b1,d–yellow dots) and concave-shaped (Fig. 2c1,d–red dots) impediments (Post-hoc Tukey’s honest significant difference (HSD) test, *Z* = 2.489, *p* = 0.034; *Z* = 3.556, *p* = 0.001, respectively). We, however, did not observe a significant difference of ***S*** between the band- and concave-shaped impediments (Post-hoc Tukey’s HSD test, *Z* = −1.067, *p* = 0.535).

### Model

To quantitatively express the hypothesis that pheromone-based processes underlie the similarity between ant behavior and optics, we built a set of simple mathematical formulae that could explain and predict the frequency distribution along the analysis region (symbols used in the model are explained in the Table S4 of Supplementary Materials). The idea behind the formulae is that initially, each foraging ant chooses the point of the passage (*y*-value in the model) when traveling to the nest and to the bait. At the early stage of foraging when there are no pheromone trails, each *y*-value has equal probability to be chosen by a foraging ant. Each foraging ant at the early stage in our model maintains its point of passage in the subsequent travels until attraction to pheromone changes the individual’s behavior. We assumed in the model that the substrate-specific walking speeds of ants are constant, resulting in trail-specific ***travel time*** (***T***) values. The model also assumes that an ant deposit a constant amount of pheromone per unit length, independently of an ant’s walking speed, the travel distance, and total travel time along that path. We thought it was a reasonable assumption considering the absence of empirical data on the possible relationships between walking speed of an individual ant and the concentration of the pheromone deposited along the trail by that ant for this species. Under these assumptions, we expected the initial rate of pheromone accumulation by foraging ants (in an experimental trial) is higher for the trails with shorter ***travel time*** (***T***) because ants that use the shorter path can travel back and forth from bait to the nest (or *vice versa*) more frequently while depositing the pheromone each time. That is, each point in the trail is more frequently visited by foraging ants if ***travel time*** is short, and this leads to faster accumulation of the pheromone on this path. The initially faster pheromone accumulation triggers a positive reinforcement mechanism: The deposited pheromone attracts even more pheromone-depositing ants, who additionally contribute to the increase of the pheromone deposit. Therefore, we assumed that after the pheromone trails are fully formed, the ***traverse frequency*** (***f***) along the trail is proportional to some power of the ***travel time***’s (***T***) inverse: $\hat{f}(y)=A{\left(\frac{1}{T(y)}\right)}^{k}$ (See **Methods** for the details). By the shape of the equation, ants crossing the concave-shaped impediment near the midline would experience the minimum travel time, and those crossing the impediment distantly from the midline would face a highly increased travel time (Fig. 1f, red line). However, for the convex-shaped impediment, ants could choose a wider range of trails near the midline without facing a highly increased travel time (Fig. 1f, blue line). Therefore, we predicted a wider dispersion of trails around the midline in convex-shaped impediment than in band- or concave-shaped ones.

By comparing the theoretical and empirical results, we found that for a specific range of *k* values (12, 13, or 14), the theoretical values of ***Ŝ*** generated for each of the 18 tests using the equations (Fig. 2e) did not differ significantly from the empirical ones (Wilcoxon signed-rank tests, *Z* = 1.023, *W* = 109, *p* = 0.306; *Z* = −0.240, *W* = 80, *p* = 0.811; *Z* = −1.372, *W* = 54, *p* = 0.170 for *k* = 12,13, and 14, respectively, see Table S3).

## Discussion

The results suggest that ants, similar to photons (rays of lights), tend to travel along time-reducing paths, which is consistent with general optimization principles^1^, laws of optics, and optimal foraging theory^2–6^. Specifically, optimal foraging theories predict that natural selection may result in the ability of animals to maximize foraging efficiency by minimizing travel time during foraging trips^4–6^. Both ant trails and light routes are the outcomes of multiple agents’ behavior (individuals or photons), and there is no central control to coordinate collective behavior. However, there is no need for a photon to ‘remember’ its previous trajectories, and physical interactions with the environment suffice to generate the time-minimizing propagation. Ants, on the other hand, utilize their own memory^19–21^ as well as the pheromone laid by other workers along the trail. The previous study^18^ hypothesized that the pheromone-based process is the underlying principle of similarity to optics. Our model grasped the core properties of trail formation, and the model results are congruent with the empirical results. The proposed model thereby fulfills two empirical conditions: it is consistent with the positive feedback process involved in pheromone trail formation, and it predicts a similarity between the observed ants’ behavior and the laws of optics.

Although the empirically observed differences in track dispersion among impediment shapes are generally consistent with the predictions from time-minimizing principles, a substantial variation was observed, and thus other factors should be considered. In tests of concave-shaped and band-shaped impediments, where the direct path was clearly time-minimizing, some ants used paths deviating from it. In the convex-shaped impediments, in contrast, some ants still used the direct path yielding a disadvantage in travel time. For the former case, the density of ants on the trail may have bolstered the dispersion as ants try to avoid collision with others^10^, or as ants tend to deviate from the pheromone path^22^. For the latter case, ants could have instinctively preferred the direct path. In nature, where the direct path is generally time-minimizing, ants could have evolved a tendency to follow the direct path. It is possible that ants integrate time-reducing and distance-reducing manners. Studies show that ants have information about the local topographies and utilize path integration, spatial and geometric cues when navigating^11–13,19^. Furthermore, ants may not only consider time or distance minimization, but also energy efficiency maximization as an important criterion in optimal path formation^23^. Thus, the actual path may be slightly different from the time-minimizing path, leading to the lack of precise agreement with the expectation based on time minimization. Scenic familiarity may have affected the dynamics of trail formation through repeated trials as well^24^. Besides, there is evidence that coordinated collective behavior is not an essential factor for the trail formation: memories of individuals may suffice to form the robust trails in certain conditions^20,21^. Moreover, ants may be trapped in suboptimal routes due to the positive feedback from pheromone deposition^9^, which could be another reason for the discordance of ant trails and predictions.

Ants also tend to follow structural edges^14,15^ which is visible in Fig. 2a1, b1, c1. We observed that some ants veer when they arrive at the Velcro impediment, and they follow along the edge before crossing into the Velcro. However, our simple model does not consider this effect when calculating time required to walk from the food to the nest. Otherwise, we assumed that the difference of travel time generated by edge-following has minimal effects on the results from an ideal model. In reality, however, we suspect that due to the shape of the impediment, the edge-following ants might be more likely to diverge from the impediment’s midline for convex-shaped impediments and converge towards midline for concave-shaped impediments. In such a situation, the edge-following behavior may affect the observed differences in the dispersion (***S***) among different impediment shapes. Therefore, in future experiments of similar design, it is necessary to measure the temporal changes in the pheromone deposition behavior, pheromone concentrations, volatility, edge-following behavior, and distribution of tracks crossing the impediment to empirically determine the mechanism responsible for the observed results. As we observed only the later stage (60–90 min after the initiation) of the trail formation process, individual movements at the early stages of trail formation must be analyzed in order to fully understand the mechanism responsible for the observed differences in the dispersion (***S***) among different impediment shapes after one hour of trail-forming activities by ants. We suggest that the index of trail dispersion, ***S***, proposed in our study, could be useful in the future research of ant trails’ formation.

In summary, this is the first empirical demonstration that the differences in spatial dispersion of trails by ants across different lens-shaped impediments are generally similar to the dispersion differences of travel-time-minimizing routes of light rays across optical lenses of different shapes. Furthermore, the observed differences can be predicted from a simple mathematical model that captures the nonlinear nature of the pheromone trail formation process. The model suggests that the similarity with optics might be a consequence of travel-time-reducing principles that govern the two entirely different processes: the collective trail formation by ants and the behavior of light across media. However, detailed empirical observations of ant behavior on impediment edges during the process of pheromone trail formation are necessary to evaluate alternative explanations for this similarity.

## Methods

### Experimental procedures

Experiments were performed at three *Camponotus japonicus* colonies (denoted by A1, MS, and MG). An average distance between colonies was 131.7 m, and they were located at Seoul National University Gwanak campus (latitude = 37° 27′ N; longitude = 126° 57′ E). We conducted trials at MS, MG in 2016 and at A1 in 2017. (The number of ants on the arena for each colony is described in Table S2) As ants from different colonies often scuffle^25^, by moving an ant from one colony to the vicinity of another colonies’ entrance, we were able to confirm that the three colonies were distinct from each other. The colonies we chose were large enough for the ants to form trails (average number of ants on the experimental arena during analysis period> 17). As the pheromone leading to the ephemeral food source evaporates relatively rapidly^26,27^, a certain minimal number of ants might be required for the trail formation that resembles optical rules (however, no information on such evaporation of pheromone for our study species exists). However, the excessively large colonies were avoided because the bait near a gigantic colony is densely covered and fringed by many ants. If so, some ants are physically marginalized from the bait and cannot participate in the ordinary foraging process.

**Figure 3:**
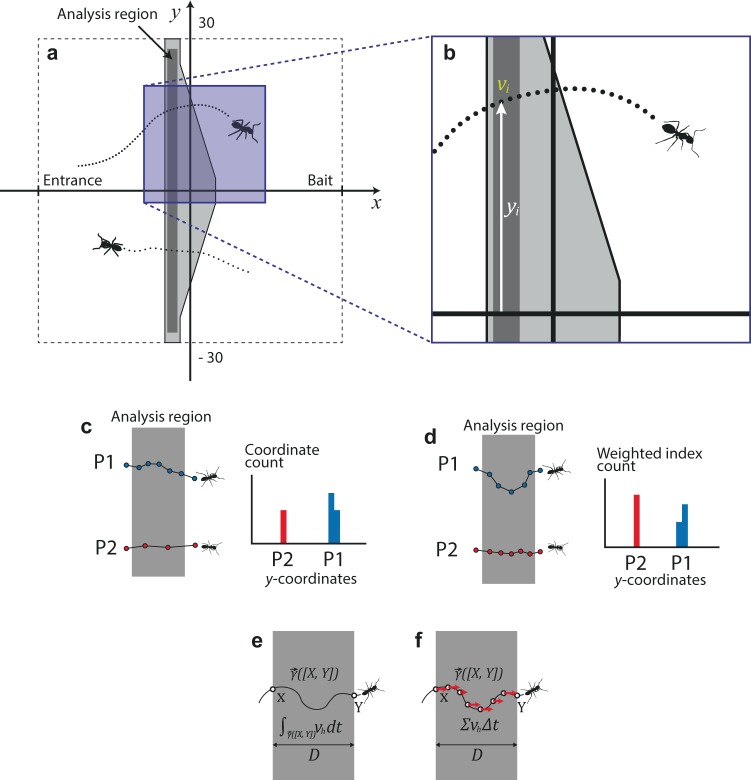
The coordinate system used for the analysis (**a,b**), and examples of two types of biases to consider in the analyses of the dispersion (**c,d**). (**a**)–The coordinate system in which *x*-axis is the midline. (**b**)–Magnified image of the blue-colored area in (**a**). As the speed and the position information is provided in a discrete manner, one can allocate speed values to the trace positions as weights: *v*_*i*_ is the speed in the *i*-th frame; *y*_*i*_ is the displacement from midline in the *i*-th frame. Both of *v*_*i*_ and *y*_*i*_ are from a specific trajectory. (**c**)– Using the sample standard deviation of *y*_*i*_’s within the analysis region as an index of dispersion is not correct. That is because the frequency distribution of *y*-values within the analysis region is biased toward slower ants, who leave more trace coordinates on the analysis region (P1, blue dots), as compared to the faster ants (P2, red dots). This is indicated by the difference of histogram area between P1 and P2 (P1 > P2). (**d**)–Using the weighted standard deviation from the distribution of *y*_*i*_’s weighted by the associated walking speed values, *v*_*i*_, is not correct either. This weighted frequency distribution of *y*-values within the analysis region is biased toward ants with longer tracks, whose sum of speed values are larger (P1; notice the larger distances between consecutive blue dots indicating the consecutive frames). This track can be compared to the relatively straight one moving in a horizontal direction (P2, red dots), which is indicated by the difference of histogram area between P1 and P2 (P1 > P2). (**e**)–The integral of horizontal speed on the analysis region is chosen as the weight in the calculations of dispersion index, ***S*** (more explanation in the text). (**f**)–Same as (**e**) for the summation.

We prepared a 100 mm-wide Velcro tape (manufactured by Dong Yang Rubber) and cut it into 3 shapes: convex, band, and concave shapes. We rigged a tripod, a camcorder, and a 600 mm × 600 mm experimental arena with one of the impediments located in the middle of the arena as shown in Fig. 1 and in Fig. 3a near a colony’s entrance. The arena was surrounded by 3-cm-high walls on which synthetic grease was daubed to hinder ants from climbing the walls.

Initially, we manually placed the ants alongside the bait and started filming right after a sufficient number (normally more than 5) of ants showed interest in the bait. We filmed for at least 90 minutes. All the videos were recorded with a 30-fps-camcorder during daytime under the shade made by nearby buildings to avoid overheating caused by direct sunlight.

The bait was composed of canned tuna with constant contents of sugar (mixture ratio of tuna to sugar = 17:1). The surface portion without the Velcro was covered with papers that were replaced on each round of an experiment to remove pheromone deposition. The Velcro was rubbed with an ethanol solution after each round of an experiment and dried for more than one day to remove the effect of the remaining pheromone.

At each colony, we performed two sets of experiments. Each set was composed of three treatments: concave-, band-, convex-shaped impediments, yielding 18 videos in total. We randomly mixed the order of the impediment in each set. The gap between the two sets in the same colony was 17.7 days on average (minimal gap: 11 days). This gap should be sufficient for the outside foragers to be replaced because the life expectancy of the outside foragers is known to be short (*e.g. P. owyhee*, 14 days^28^; *C. bicolor*, 6.1 days^29^). The average duration of each set was 5 days.

### Video analysis and the *data matrix*

Using Adobe Premiere Pro CC, we adjusted contrast and brightness to facilitate the tracking efficiency by the video tracking software AnTracks (http://www.antracks.org). The arena in the video was adjusted into the square shape to correct the distortion caused by camera alignment. If an ant stood stationary for more than 5 s on the analysis region, we removed the corresponding points from the analysis by covering the points with a white layer. In that way, we exclusively analyzed mobile foraging ants. Images from the videos that are evidently not ants such as dirt, other insects, or shades of Velcro fibers were also erased from video by applying such layers.

We analyzed the edited videos from 60 to 90 min (30-min excerpt) after the initiation of filming when the trails were assumed to be stabilized. The video was transported to AnTracks to extract positions of ants in each frame. AnTracks provided the positions, trajectory IDs, time, and other information about ants’ movement. Then, we used MATLAB R2017a to analyze the extracted coordinates.

Firstly, we excluded positions of ants with walking speeds outside the range between 1.5 cm/s and 20 cm/s. Walking slower than 1.5 cm/s were considered to be stationary, and objects which moved faster than 20 cm/s were considered erratic (they might have been flying insects). We also excluded trajectories whose duration is less than 0.5 s as extremely short trajectories are likely to be the traces of temporary shades or other flying insects.

We generated ‘heatmaps’ to visualize the distribution of walking ants on the whole experimental arena. Each heatmap is composed of 600 × 600 grids. As the side length of the experimental arena is 600 mm, a grid in a heatmap represents 1 mm × 1 mm area. Slower walking ants generate more coordinates during tracing and are more likely to contribute to more grids. In order to remove this bias, the contribution of a mobile ant to a grid should increase proportionately to the walking speed. Hence, each record of an ant in a grid was weighted by the corresponding walking speed at each position. The brighter grid color (higher grid values), therefore, indicates that ants were more likely to cross that grid. Examples of heatmaps are shown in Fig. 2a1, b1,c1.

The statistical analysis was conducted on the data recorded from a narrow analysis region (Fig. 3). Consecutive frame-by-frame positions (*x*, *y* coordinates) of the same ant within the analysis region form a trajectory. This enabled us to calculate the walking speed at each position. From the distance an ant moved between two consecutive frames (Δ*t* =1/30 s), we calculated the walking speed of each position in a trajectory as ${v}_{i}=|({x}_{i+1},{y}_{i+1})-({x}_{i},{y}_{i})|/\Delta t$. In a similar fashion, we calculated the horizontal speed (*i.e*. the norm of the velocity vector’s horizontal component which is parallel to the midline or *x*-axis in Fig. 3a,b) according to the formula ${v}_{i,h}=|{x}_{i+1}-\,{x}_{i}|/\Delta t$. For each of the 18 tests, we put data from all ant tracks into a ***data matrix*** containing two columns of coordinates, one column of speed values, and one column of horizontal speed values for all ant records in a test. This data was then subjected to analysis in order to extract a variable representing the degree of dispersion of foraging ants in each of the 18 tests.

### Data analysis: an overview

We used the ***data matrix*** to determine how much the trajectories of ants traversing the impediment deviated from the midline. Let *v*_*i*_ denote the speed of *i*-th traced coordinate and *y*_*i*_ the *i*-th displacement from the midline. *v*_*i*_ and *y*_*i*_ are from the ***data matrix*** (Fig. 3a,b). In order to measure the dispersion, we decided to use the weighted standard deviation of *y*_*i*_’s within the analysis region (we refer to it as the index of dispersion, *S*). Weights of *y*_*i*_’s are horizontal speed associated with each data point. We used horizontal speed because the number of points per track within the analysis region is larger for slowly moving ants than that of fast-moving ants. Ants with longer trails on the analysis region also have a similar bias. If all of the *y*_*i*_’s coordinates in the *data matrix* have the same weight when calculating the standard deviation of *y*_*i*_’s, the contribution of ants with longer trails or slow speed to the ***S*** (*e.g*. P1 in Fig. 3c) would be disproportionately larger than the contribution of ants with shorter trails or fast speed (*e*.*g*. P2 in Fig. 3c). It is, however, not sufficient to weight the distribution by the speed value to remove this bias as indicated by Fig. 3d. We, therefore, have multiplied the value of *y*-coordinates by the horizontal speed value.

In this way, even though the slow and longer tracks contribute a large number of data points, each of the data points is weighted less than each of the few data points of fast and straight tracks. By this measure, each track contributes equally to the total frequency distribution regardless of the walking speed, distance traveled, and turning frequency while traversing the analysis region. Therefore, this distribution is similar to the relative distribution of the number of tracks crossing the analysis region at various displacements (*y*) from the midline. Examples of the weighted frequency distributions are shown in Fig. 2. Finally, we calculated weighted standard deviation ***S*** as an index of dispersion of ant tracks crossing the analysis region. Small values of ***S*** indicate the convergence of trails on the impediment, and large values indicate the divergence. Mathematical formulae and details are described in the following section.

After obtaining ***S*** for each test, we used the linear mixed-effects models to evaluate the effect of the impediment shape on the ***S*** using the *lmer* function (the package lme4 in R (Bates *et al*. 2015)). In the model, the nest ID and the set ID were considered as random effects. When comparing mathematical expectations and empirical results, we used the Wilcoxon signed-rank test (in MATLAB R2017a).

### Data analysis: mathematical calculations to obtain the index of dispersion, S

As the fidelity of tracking is not perfect for small ants, the traced trajectories of some ants are disconnected and fragmented. When using an analysis line rather than an analysis region for the count of traverses, the trajectories of ants whose segments do not cross the analysis line will not be counted. When adopting an analysis region, though some traces are fragmented, each segment can contribute to the overall results as long as the segment is contained in the analysis region. Therefore, utilizing the analysis region is relatively robust against tracking fidelity and continuity problems while producing similar results to the coordinate count on the line.

Let *v*_*i,h*_ denote the horizontal speed of *i*-th traced coordinate. Also, let $\overrightarrow{\gamma }([X,Y])$ denote the trajectory of an ant that links one point on the boundary of analysis region (*X*) and another point on the other side of the boundary (*Y*). $\overrightarrow{\gamma }([X,Y])$ is theoretically continuous but discrete in an empirical sense, because it was measured in each frame whose duration is 1/30 s. As ants rarely turn backward on the impediment, we assumed that the sign of the horizontal velocity remains unchanged. We did not use the speed between two consecutive frames as weights because by doing so, we would have introduced a bias: ants with longer trajectories would have become overrepresented in the frequency distribution of *y*-values (Fig. 3d). Mathematically speaking, let *φ*_*j*_ be the set of speed values of $\overrightarrow{{\gamma }_{j}}([X,Y])$, then,

1 $${\sum }_{{v}_{i}\in {\varphi }_{j}}{v}_{i}\approx L/\Delta t$$

where *L* is the distance an ant moved on the analysis region in a single (convoluted) trajectory, and Δ*t* is a duration of a frame (1/30 s). This is derived from the simple equation that $\sum {v}_{i}\Delta t$ equals the distance that an ant moved. As Δ*t* is constant, the summation of the speed values on the analysis region is in proportion to the actual distance on which the ant moved. Accordingly, multiplying *y*_*i*_ by the *v*_*i*_ as the weight will produce biased results: For instance, if an ant curved its path on the analysis region as P1 in Fig. 3d, its distance would increase though both ants traversed the analysis region once.

To avoid these problems, we used horizontal speed values (*v*_*h*_) as the weight associated with *y*_*i*_’s. Unlike the aforementioned methods, whichever paths ants take through the impediment, the sum of the horizontal movement in a single passage is equivalent to the width of the impediment, given that there is no turning back. In other words,

2 $${\int }_{\overrightarrow{\gamma }([X,Y])}{v}_{h}dt=D$$

where *D* is the width of the analysis region. Thus, horizontal speed on the $\overrightarrow{\gamma }([X,Y])$ is an appropriate measure that is robust against turns and walking speed.

Similarly, as the stepwise version of the equation, the following similarity holds (Fig. 3e,f).

3 $${\sum }_{{v}_{i,h}\in {\varphi }_{j}}{v}_{i,h}\Delta t\approx D$$

where *v*_*i,h*_ (*v*_*i,h*_ ≥ 0) is the horizontal speed in the *i*-th frame in a specific trajectory, and *φ*_*j*_ is the set of horizontal speed values in $\overrightarrow{\gamma }([X,Y])$. Therefore, once the sign of the horizontal velocity is maintained, whichever direction and speed an ant exhibits, the sum of horizontal speed values is nearly constant for every traverse. This property allowed us to adopt horizontal speed as the weight for building the frequency distribution of *y*-values, from which the dispersion index, ***S***, was derived. Examples of such weighted frequency distributions are shown in Fig. 2.

Hence, the index of dispersion used for the analysis is the weighted standard deviation of the ants’ *y*-positions on the impediment weighted by their horizontal speeds, that is,

4 $${\boldsymbol{S}}={\left(\frac{{\sum }_{i=1}^{{N}_{a}}{v}_{i,h}{({y}_{i}-\overline{{Y}_{h}})}^{2}}{\frac{{N}_{a}-1}{{N}_{a}}{\sum }_{i=1}^{{N}_{a}}{v}_{i,h}}\right)}^{1/2}$$

where $\overline{{Y}_{h}}$ is the weighted mean of *y*_*i*_’s $(\overline{{Y}_{h}}={\sum }_{i}{v}_{i,h}{y}_{i}/{\sum }_{i}{v}_{i,h})$. All *y*_*i*_’s are included in the analysis region with non-zero horizontal speed values and *N*_*a*_ is the total number of such *y*_*i*_’s.

This index gets larger as more ants generate dispersing paths. We used this index to compare dispersion among experimental treatments.

### Description of formulae

As many of the ants passing through the Velcro moved nearly horizontally, and for simplicity of the model, ants are assumed to travel horizontally on the impediment. Ants were also assumed to travel directly in a straight line toward the entrance, bait, or edge of the impediment while on the paper-lined surface (Fig. 1e). Accordingly, the shape and configuration of the path are totally dependent on the *y*-coordinate of the passage point (equivalent to the *y*_*i*_’s in Fig. 3). As long as the speeds on the paper and on the impediment are fixed, the total travel time is determined by the distance an ant moves on each surface divided by the corresponding speed.

5 $$T(y)=\frac{{L}_{N}(y)}{{v}_{N}}+\frac{{L}_{V}(y)}{{v}_{V}}$$

where *L*_*N*_(*y*) and *L*_*V*_(*y*) are the distances an ant needs to travel on the paper-lined and on the Velcro surface (*N* stands for normal; *V* for Velcro), respectively; *v*_*N*_ and *v*_*V*_ are the speed values on the paper-lined surface and on the Velcro surface, respectively. Speed values in this process are assumed to be constant.

Assuming constant speed, *T*(*y*) can be written as follows.

6 $$T(y)=\frac{1}{{v}_{N}}\left(\frac{{L}_{N}(y)}{1}+\frac{{L}_{V}(y)}{\frac{{v}_{V}}{{v}_{N}}}\right)=\frac{1}{{v}_{N}}\left(\frac{{L}_{N}(y)}{1}+\frac{{L}_{V}(y)}{{v}_{r}}\right)$$

where *v*_*r*_ is the relative ratio of *v*_*V*_ to *v*_*N*_.

The formation of pheromone trails by ants relies on positive feedback between the number of the ants using the trail and the amount of the pheromone, which exhibits nonlinear relationship^30,31^. As the first-line design of the formulae to embrace these facts, we assumed that the relative frequency density along the impediment’s *y*-coordinates is in proportion to the *k*-th power of 1/*T*(*y*). In this way, the longer traveling time induces weaker trails, while the shorter traveling time induces even stronger trails. One can accordingly derive the relative frequency of the traverse across the analysis region as follows:

7 $$\psi (y)=\frac{1/{T}^{k}(y)}{{\int }_{-{y}_{0}}^{{y}_{0}}\frac{1}{{T}^{k}(y)}dy}$$

where *y*_0_ = 28 *cm* in this study. As $\frac{1}{{v}_{N}}$ is canceled out in *ψ*(*y*), one can notice that the speed does not affect the relative frequency as long as *v*_*r*_ is constant. In other words, whichever *v*_*N*_ one chooses, the expected relative frequency of the passage positions is invariant.

For each of the 18 empirical tests, we calculated the theoretically expected degrees of dispersion (***Ŝ***) for the 20 values of *k* from 1 to 20 in steps of 1. Thereby, ***Ŝ*** can be derived from the following equation.

8 $$\hat{{\boldsymbol{S}}}={\left(\frac{{\int }_{-28}^{28}{(y-\bar{Y})}^{2}\psi (y)dy}{{\int }_{-28}^{28}\psi (y)dy}\right)}^{\frac{1}{2}}={({\int }_{-28}^{28}{y}^{2}\psi (y)dy)}^{\frac{1}{2}}$$

This equation represents the method for deriving standard deviation from a continuous variable. ${\int }_{-28}^{28}\psi (y)\,dy=1$ by the definition of *ψ*. The weighted mean $\bar{Y}=0$ due to the symmetry of *ψ*. For each value of *k*, we compared the 18 expected values of ***Ŝ*** with the 18 empirically observed values of ***S***. The high *p*-values derived from the Wilcoxon signed-rank test (Table S3) indicate that no significant difference was observed between theoretical and empirical observations when *k* = 12, 13, and 14.

## Supplementary information

Supplementary Materials
