# Supplementary material for: Trails of ants converge or diverge through lens-shaped impediments, resembling principles of optics
Source: Sci Rep. 2020 May 21;10:8479. doi: 10.1038/s41598-020-65245-0 (PMC7242390; doi:10.1038/s41598-020-65245-0)
Supplement: Supplementary file 1 — Supplementary Materials [file 41598_2020_65245_MOESM1_ESM.docx]

Supplementary Materials

**Supplementary Materials to: “Trails of ants converge or diverge through lens-shaped impediments, resembling principles of optics”**

Jibeom Choi^1^, Hangah Lim^1^, Woncheol Song^1^, Han Cho^2^, Ho-Young Kim^3*^, Sang-im Lee^4*^, Piotr G. Jablonski^1,5*^

^1^Laboratory of Behavioral Ecology and Evolution, School of Biological Sciences, Seoul National University, Seoul 08826, South Korea

^2^College of Medicine, Seoul National University, Seoul 03080, South Korea

^3^Department of Mechanical and Aerospace Engineering, Seoul National University, Seoul 08826, South Korea

^4^School of Undergraduate Studies, Daegu-Gyeongbuk Institute of Science and Technology, Daegu 42988, South Korea.

^5^Museum and Institute of Zoology, Polish Academy of Sciences, Warsaw 00-679, Poland

*Corresponding Authors: Piotr G. Jablonski ([piotrjab@behecolpiotrsangim.org](mailto:piotrjab@behecolpiotrsangim.org)), Sang-im Lee ([sangim@dgist.ac.kr](mailto:sangim@dgist.ac.kr)) & Ho-Young Kim ([hyk@snu.ac.kr](mailto:hyk@snu.ac.kr))

**SUPPLEMENTARY RESULTS**

**Measuring the speed and density of ants**

From the start of a recording to 15 min in each video, we manually traced the position of ants to achieve the actual speed using MaxTRAQ software. (For one video, due to the lack of ants in the early stage of trail formation, we traced from the start to 30 min.) As ants frequently stop or turn, we chose the fragment of paths that are relatively straight. Among them, we chose those that traversed Velcro impediment and paper-lined normal surface. We selected 5 outbound (toward bait) and 5 inbound (toward entrance) ant paths in each video and traced their speed on the normal surface (covered by paper) and on Velcro. After frame-by-frame (duration of a frame = 0.1 s) manual digitization, we obtained average speed on the normal surface and on Velcro. The speed on both substrates, deceleration ratio ($v_{r}$) of each Set, colony, and the impediment are shown in **Table S1**. These deceleration ratios were used to calculate the theoretically predicted relative frequency distributions in the mathematical model.

The mean walking speed on the normal surface (mean ± SD; 8.7 ± 4.3 cm/s, *n* = 180) was significantly higher than the speed on the Velcro surface (4.2 ± 2.0 cm/s, *n* = 180; Wilcoxon signed-rank test, *Z* = 6.22, *W* = 12125, *p* < 0.0001). We also observed that the speed on the normal surface in Set 1 (9.5 ± 4.7 cm/s, *n* = 90) is significantly higher than the speed in Set 2 (7.8 ± 3.7 cm/s, *n* = 90; Mann-Whitney *U* test, *Z* = 2.61, *p* < 0.01) presumably due to the temperature difference. The same was true for walking speeds on the Velcro (Set 1: 4.7 ± 2.2 cm/s, *n* = 90; Set 2: 3.8 ± 1.6 cm/s, *n* = 90; Mann-Whitney *U* test, *Z* = 2.84, *p* < 0.01). According to Korea Meteorological Administration^[[1]](#endnote-2)^, the average temperature of Seoul, the Republic of Korea when experiments of Set 1 were performed (29.5 ± 1.3℃, *n* = 9) was significantly higher than the average temperature when experiments of Set 2 were performed (24.7 ± 2.4℃, *n* = 9; Mann-Whitney *U* test, *p* < 0.001, *Z* is not provided). This is compatible with previous observations that the moving speed of ants positively correlates with temperature^[[2]](#endnote-3),^^[[3]](#endnote-4)^.

To measure the number of ants on the arena, we counted the number of ants which adhered to the bait (referred to as core ants) and the ants which did not (referred to as marginal ants). We counted at 60, 70, 80 and 90 min from the initiation of recording and calculated an average from these four counts as an index of colony size (**Table S2**). We inferred that the colony MG is the biggest, and the colony A1 is the smallest.

The index of dispersion (***S***) for each experiment, which is presented in **Fig. 2d** in the main text, are additionally shown in **Fig. S1,** where they are organized by the colony name.

**Model Results**

By utilizing empirical speed values obtained from video analysis, we built equations that expect travel time by traverse position (**Fig. S2**). The traverse position in the model indicates the *y*-values on the analysis region (See **Fig. 3b**). As explained in the main text, we could not find a significant difference between empirical results and theoretical results for specific values of *k* in the model (**Table S3**).

|  | Variable | Convex-shaped | Band-shaped | Concave-shaped | Total |
| --- | --- | --- | --- | --- | --- |
| A1 Set 1 | $\boldsymbol{v}_{\boldsymbol{N}}$ (cm/s)  $\boldsymbol{v}_{\boldsymbol{V}}$ (cm/s)  $\boldsymbol{v}_{\boldsymbol{r}}$ | 4.5 ± 1.0  3.4 ± 1.0  0.76 ± 0.1 | 3.9 ± 1.0  2.5 ± 1.1  0.62 ± 0.1 | 3.6 ± 0.7  2.7 ± 0.5  0.77 ± 0.1 | 4.0 ± 0.9  2.9 ± 0.9  0.71 ± 0.1 |
| A1 Set 2 | $\boldsymbol{v}_{\boldsymbol{N}}$ (cm/s)  $\boldsymbol{v}_{\boldsymbol{V}}$ (cm/s)  $\boldsymbol{v}_{\boldsymbol{r}}$ | 4.1 ± 1.2  3.2 ± 1.2  0.76 ± 0.1 | 3.3 ± 0.6  2.6 ± 0.7  0.78 ± 0.1 | 3.7 ± 0.8  2.6 ± 0.7  0.69 ± 0.1 | 3.7 ± 0.9  2.8 ± 0.9  0.74 ± 0.1 |
| MS Set 1 | $\boldsymbol{v}_{\boldsymbol{N}}$ (cm/s)  $\boldsymbol{v}_{\boldsymbol{V}}$ (cm/s)  $\boldsymbol{v}_{\boldsymbol{r}}$ | 11.3 ± 2.2  5.1 ± 1.4  0.47 ± 0.1 | 10.3 ± 3.6  4.0 ± 1.7  0.39 ± 0.1 | 13.5 ± 3.1  5.6 ± 1.8  0.42 ± 0.1 | 11.7 ± 3.2  4.9 ± 1.7  0.43 ± 0.1 |
| MS Set 2 | $\boldsymbol{v}_{\boldsymbol{N}}$ (cm/s)  $\boldsymbol{v}_{\boldsymbol{V}}$ (cm/s)  $\boldsymbol{v}_{\boldsymbol{r}}$ | 10.0 ± 1.3  3.6 ± 0.7  0.35 ± 0.2 | 7.5 ± 1.7  2.9 ± 0.9  0.39 ± 0.1 | 10.2 ± 1.0  4.4 ± 1.4  0.43 ± 0.1 | 9.2 ± 1.8  3.6 ± 1.2  0.39 ± 0.1 |
| MG Set 1 | $\boldsymbol{v}_{\boldsymbol{N}}$ (cm/s)  $\boldsymbol{v}_{\boldsymbol{V}}$ (cm/s)  $\boldsymbol{v}_{\boldsymbol{r}}$ | 12.4 ± 1.7  5.5 ± 1.4  0.44 ± 0.1 | 12.6 ± 4.1  6.7 ± 3.0  0.52 ± 0.1 | 13.4 ± 2.9  6.7 ± 2.1  0.50 ± 0.1 | 12.9 ± 3.0  6.3 ± 2.3  0.49 ± 0.1 |
| MG Set 2 | $\boldsymbol{v}_{\boldsymbol{N}}$ (cm/s)  $\boldsymbol{v}_{\boldsymbol{V}}$ (cm/s)  $\boldsymbol{v}_{\boldsymbol{r}}$ | 11.9 ± 4.5  5.8 ± 2.2  0.50 ± 0.1 | 8.3 ± 2.1  3.7 ± 1.5  0.43 ± 0.1 | 11.3 ± 1.7  5.2 ± 1.2  0.47 ± 0.1 | 11.3 ± 3.3  5.2 ± 1.9  0.46 ± 0.1 |

**Table S1. The speed (cm/s) of ants walking on the paper-lined surface (**$\boldsymbol{v}_{\boldsymbol{N}}$**), Velcro (**$\boldsymbol{v}_{\boldsymbol{V}}$**), and deceleration ratios (**$\boldsymbol{v}_{\boldsymbol{r}}$ **=** $\boldsymbol{v}_{\boldsymbol{V}}$**/**$\boldsymbol{v}_{\boldsymbol{N}}$**) across convex-shape, band-shaped, and concave-shaped impediments in each Set and the colony (average ± SD).** Note that the average deceleration ratio is the average of individuals’ respective deceleration ratios. Therefore, it may not be the same as average speed on the Velcro divided by that on the paper-lined surface. For example, on the convex-shaped impediment of MS Set1, $\frac{\bar{v_{V}}}{\bar{v_{N}}}=\frac{5.1}{11.3}\approx0.45\neq0.47=\bar{v_{r}}$. Speeds were measured for walking ants (*n* = 10) in each experiment. As ants frequently halt, the general average speed of all foraging ants measured over the whole duration of their presence in the video clip, regardless of whether they walk or not, would have been lower than the values presented here.

|  | |  | | **A1** | | **MS** | | **MG** | |  |
| --- | --- | --- | --- | --- | --- | --- | --- | --- | --- | --- |
|  |  | | Marginal | | Core | Marginal | Core | Marginal | Core | |
| Convex | Set 1 | | 11.0 | | 18.0 | 21.0 | 21.5 | 72.8 | 48.3 | |
|  | Set 2 | | 6.0 | | 14.3 | 27.8 | 39.8 | 44.8 | 64.0 | |
| Band | Set 1 | | 10.3 | | 30.0 | 22.0 | 29.8 | 72.8 | 32.0 | |
|  | Set 2 | | 4.3 | | 13.3 | 23.3 | 53.8 | 49.8 | 68.3 | |
| Concave | Set 1 | | 7.5 | | 21.3 | 16.8 | 16.5 | 98.3 | 48.8 | |
|  | Set 2 | | 11.3 | | 27.8 | 27.0 | 50.8 | 81.0 | 51.5 | |
| Average |  | | 8.4 | | 20.8 | 23.0 | 35.3 | 69.9 | 52.1 | |
| Total average (marg. + core) |  | | **29.1** | | | **58.3** | | **122.0** | | |


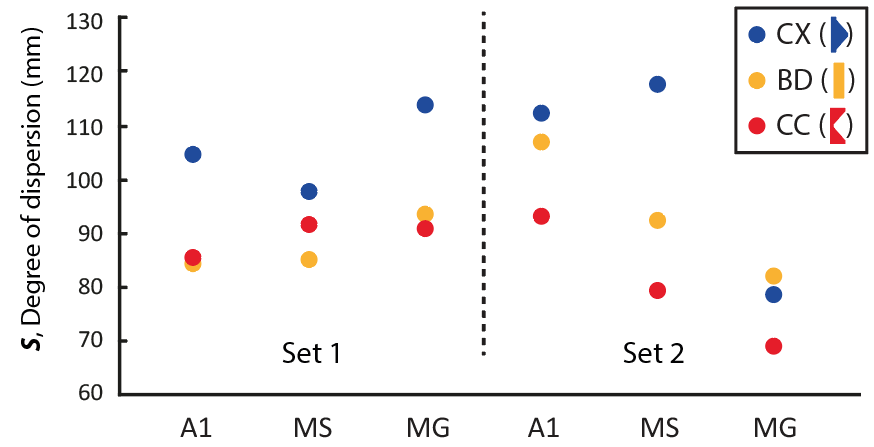


**Table S2. The average number of individuals on the foraging arena in convex-shape, band-shaped, and concave-shaped impediment treatments in each Set (1 or 2) of experiments in each of three colonies (A1, MS, MG).**

Core ants are those at the food source; Marginal ants are the remaining ants on the experimental arena.

**Figure S1. Indices of dispersion (*S*) for experiments in Set 1 (left; average temperature (mean ± SD): 29.5 ± 1.3℃) or Set 2 (right; average temperature: 24.7 ± 2.4℃) in experiments with convex-shaped (CX, blue dots), band-shaped (BD, yellow dots), and concave-shaped (CC, red dots) impediments.**

The same results are shown in Fig. 2 of the main text in a different manner.


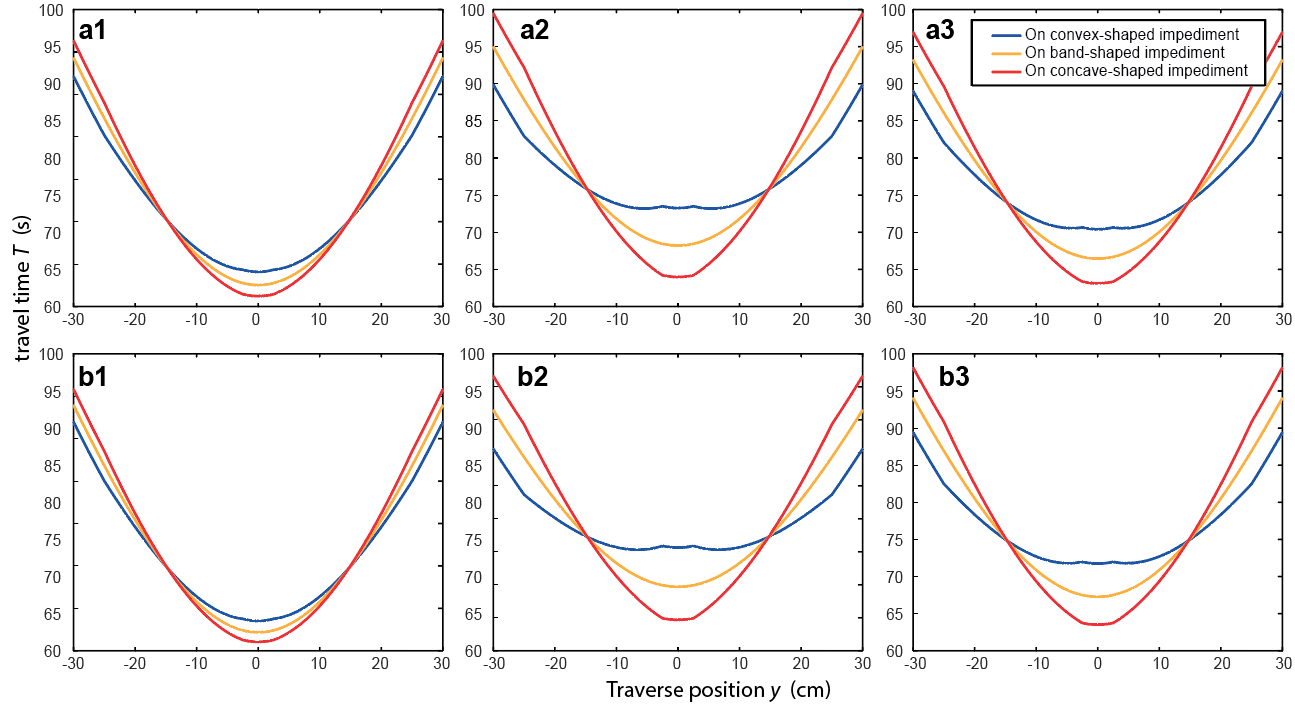


**Figure S2. Relationships between the *displacement* (or *traverse position*) from the midline (Midline is marked with value 0 on the *x*-axis.) and *travel time* (*T*) calculated from empirical deceleration ratios in each of the 18 tests.**

The first row (a1, a2, a3) represents Set 1; The second row (b1, b2, b3) represents Set 2. Each of the first (a1, b1), the second (a2, b2), and the third (A3, B3) column represents colony A1, MS, and MG, respectively. In all graphs, *v_N_* is 1 cm/s. As we utilized the data from *y* = -28 cm to *y* = 28 in empirical analysis, we also utilized the same range of *y* when analyzing theoretical data.

| power | *p*-value | power | *p*-value |  | power | *p*-value | power | *p*-value |
| --- | --- | --- | --- | --- | --- | --- | --- | --- |
| 1 | 0.0002 | 6 | 0.0002 |  | 11 | 0.0222 | 16 | 0.0108 |
| 2 | 0.0002 | 7 | 0.0002 |  | **12** | **0.3061** | 17 | 0.0057 |
| 3 | 0.0002 | 8 | 0.0002 |  | **13** | **0.7439** | 18 | 0.0038 |
| 4 | 0.0002 | 9 | 0.0003 |  | **14** | **0.1570** | 19 | 0.0025 |
| 5 | 0.0002 | 10 | 0.0014 |  | 15 | 0.0347 | 20 | 0.0018 |

**Table S3. The comparison of *S* between empirical results and theoretical expectations: results of the Wilcoxon signed-rank test.**

**Table S4. List of variables used in this study.**

| Variable | Description |
| --- | --- |
| *S* | The weighted standard deviation of *y*’s (weighted by the corresponding horizontal speed, $v_{h})$ which was used as an index of dispersion |
| $\hat{\boldsymbol{S}}$ | The theoretical weighted standard deviation of *y*’s. |
| $\boldsymbol{y}$ or $\boldsymbol{y}_{\mathbf{i}}$ | The displacement of vertical components from the midline (*y* = 0). The subscript *i* refers to its order in the set. |
| $\boldsymbol{v}$ or $\boldsymbol{v}_{\mathbf{i}}$ | The speed of an ant (in the direction of movement). |
| $\boldsymbol{v}_{\boldsymbol{h}}$ | The *horizontal* (within our coordinate system, *i*.*e*. parallel to the *x*-axis) component of the speed of an ant |
| $\boldsymbol{L}$ | The distance an ant moved on the analysis region |
| $\boldsymbol{D}$ | The width of the analysis region. |
| $\bar{\boldsymbol{Y}}$ | The weighted average of *y*. |
| $\boldsymbol{\varphi}_{\boldsymbol{j}}$ | The set of speed values in a trajectory. |
| $\vec{\boldsymbol{\gamma}}\boldsymbol{(}\left[ \boldsymbol{X,Y} \right]\boldsymbol{)}$ | The trajectory of an ant that moves from *X* to *Y*. *X* and *Y* are in the other boundary of the analysis region. |
| $\boldsymbol{L}_{\boldsymbol{N}}\boldsymbol{(y)}$ and $\boldsymbol{L}_{\boldsymbol{V}}\boldsymbol{(y)}$ | The length trajectory on paper-lined ($L_{N}$) and Velcro surface ($L_{V}$) which is determined by traverse position (*y*). |
| $\boldsymbol{v}_{\boldsymbol{N}}$ and $\boldsymbol{v}_{\boldsymbol{V}}$ | The speed on the paper-lined surface ($v_{N}$) and on the Velcro surface ($v_{V}$). |
| $\boldsymbol{v}_{\boldsymbol{r}}$ | The deceleration ratio; the ratio of $v_{V}$ to $v_{N}$. |
| $\boldsymbol{T(y)}$ | The time it takes for an ant to travel from the entrance to bait (or bait to the entrance) which is determined by traverse position (*y*). |
| $\boldsymbol{\psi}\left( \boldsymbol{y} \right)$ | The theoretical frequency of traverse through *y*. |
| $\boldsymbol{N}_{\boldsymbol{a}}$ | The number of coordinates that are included in the analysis region with nonzero horizontal speed. |

**REFERENCES**

1. Korea Meteorological Administration website, http://www.kma.go.kr [↑](#endnote-ref-2)
2. Marsh, A. C. (1985). Microclimatic factors influencing foraging patterns and success of the thermophilic desert ant, *Ocymyrmex barbiger*. *Insectes Sociaux*, *32*(3), 286-296. [↑](#endnote-ref-3)
3. Jayatilaka, P., Narendra, A., Reid, S. F., Cooper, P., & Zeil, J. (2011). Different effects of temperature on foraging activity schedules in sympatric *Myrmecia* ants. *Journal of Experimental Biology*, *214*(16), 2730-2738. [↑](#endnote-ref-4)
